# STABILITY OF COGNITIVE ABILITY AND EFFECT OF EDUCATION ACROSS 58 YEARS: A PROJECT TALENT AGING STUDY

**DOI:** 10.1093/geroni/igae098.2076

**Published:** 2024-12-31

**Authors:** Thalida Em Arpawong, Jimi Huh, Tara Gruenewald, Gwenith Fisher, Jennifer Manly, Dominika Seblova, Kelly Peters, Margaret Gatz

**Affiliations:** University of Southern California, Los Angeles, California, United States; University of Southern California, Los Angeles, California, United States; Chapman University, Orange, California, United States; Colorado State University, Fort Collins, Colorado, United States; Columbia University, New York, New York, United States; Charles University, Prague, Hlavni mesto Praha, Czech Republic; American Institutes for Research, Arlington, Virginia, United States; University of Southern California, Los Angeles, California, United States

## Abstract

We assessed the degree to which individuals retain cognitive abilities from adolescence through older adulthood, and whether more education associates with better retention in some more than others, after adjusting for adolescent cognitive ability. We also evaluated whether benefits associated with education differ for those with lower or higher cognitive scores in adolescence, or women or men. Data came from the Project Talent Aging Study, and included measures of abstract reasoning, mathematics, visualization in two dimensions, and recognition memory in 1960 and 2018 (n=2,032; age range 71 to 79). Confirmatory factor analysis was used to fit one-factor (cognitive ability factor, CAF) at each timepoint. We then used a structural equation model to test whether the 1960 CAF predicted the 2018 CAF, with an interaction term between 1960 CAF and years of education. Multiple group models tested invariance by sex. We found high stability in CAFs assessed 58 years apart. Education predicted higher 2018 CAF (p<.001), although a significant interaction (1960 CAF*education; p<.05) showed that effects were diminished for those with higher 1960 CAF. Sex-specific analyses showed that the diminishing effect of education was evident for both women (p<.05) and men (p<.05). Additionally, the simple effect of education on 2018 CAF was significant for women (p<.001) but not for men (p=.37). Findings indicate high stability in cognitive abilities from adolescence to older adulthood, and that exposure to more education differentially benefits later age abilities depending on adolescent levels and sex.

